# Validation of the Investigator Global Assessment of Chronic Hand Eczema (IGA–CHE): a new clinician reported outcome measure of CHE severity

**DOI:** 10.1007/s00403-024-02818-3

**Published:** 2024-03-20

**Authors:** Jonathan I. Silverberg, Tove Agner, Keith Baranowski, Ursula Plohberger, Henrik Thoning, Rob Arbuckle, Laura Grant, George Skingley, Robert Bissonnette

**Affiliations:** 1 School of Medicine and Health Sciences, The George Washington University, Washington, DC USA; 2 Department of Dermatology, Bispebjerg Hospital, University of Copenhagen, Copenhagen, Denmark; 3 LEO Pharma A/S, Ballerup, Denmark; 4 Patient-Centered Outcomes, Adelphi Values Ltd, Bollington, Cheshire UK; 5 Innovaderm Research, Montreal, QC Canada

**Keywords:** Chronic Hand Eczema, Contact dermatitis, Clinician-reported Outcome (ClinRO), Delgocitinib cream, Investigator global assessment (IGA), Topical pan-JAK inhibitor

## Abstract

The Investigator Global Assessment of Chronic Hand Eczema (IGA–CHE) is a novel Clinician-Reported Outcome measure that allows investigators to assess cross-sectional CHE global disease severity using clinical characteristics of erythema, scaling, lichenification/hyperkeratosis, vesiculation, oedema, and fissures as guidelines for overall severity assessment. This study aimed to evaluate the psychometric properties of the IGA–CHE for use as an outcome measure in CHE clinical trials and clinical practice. Psychometric analyses were performed using data from a sample of 280 patients with moderate to severe CHE from a phase 3 trial of delgocitinib cream, pooled across treatment groups. Test–retest reliability results were moderate to strong with kappa coefficients ranging from 0.63 to 0.76. Correlations with measures assessing related concepts were moderate or strong (range 0.65–0.72) and exceeded a priori hypotheses, providing evidence of convergent validity. Known-groups validity was supported by statistically significant differences between severity groups (< 0.001). Within-group effect sizes were consistently larger for improved groups compared to stable groups, providing evidence of ability to detect change. Anchor-based analyses generated within-subject meaningful change estimates ranging from – 0.8 to – 2.3. A correlation weighted average suggested a single value of – 1.7 in change from baseline. These findings provide evidence the IGA–CHE scale has strong reliability, construct validity, and ability to detect change, supporting its use as an endpoint in CHE clinical trials and clinical practice. Based on the evidence, 2-level changes in IGA–CHE score are considered a conservative meaningful change threshold; however, findings also indicate 1-level change in IGA–CHE scores reflects a clinically meaningful improvement for patients.

*Clinical trial registration*: NCT04871711.

**Supplementary Information:**

The online version contains supplementary material available at 10.1007/s00403-024-02818-3.

## Introduction

Chronic Hand Eczema (CHE) is one of the most frequent chronic inflammatory diseases affecting the hands [1], often caused by contact dermatitis and characterized by poor prognosis [2]. CHE refers to hand eczema that persists for more than 3 months or that returned at least twice within the last 12 months [3]. Currently, there are no topical treatments specifically developed and approved for use in CHE [3]. For trials supporting new drug registrations in dermatological conditions (e.g., atopic dermatitis), regulatory authorities have recommended that an Investigator Global Assessment (IGA) is included as a primary endpoint [4, 5]. An IGA of CHE severity that is valid, reliable, and sensitive to changes over time is, therefore, required to support evaluation of the efficacy of potential treatments for CHE. Such a measure may also be of value as a quick, easily administered method of assessing patient outcomes in clinical practice.

The Investigator Global Assessment of Chronic Hand Eczema (IGA–CHE) is a Clinician-Reported Outcome (ClinRO) measure that allows investigators to assess global disease severity at one given timepoint using clinical characteristics of erythema, scaling, lichenification/hyperkeratosis, vesiculation, oedema, and fissures to guide the overall severity assessment [6]. The IGA–CHE was originally developed by clinical experts, in line with regulatory guidance [7–11], and included in a phase 2b trial (NCT03683719) assessing the efficacy and safety of delgocitinib cream in adult patients with mild to severe CHE [12]. Subsequently, based on regulatory feedback, modifications were made to the IGA–CHE to ensure clear distinction between the severity levels in the descriptions provided. This included updating the definition of ‘almost clear’ from ‘faint erythema’ and ‘no signs of scaling, hyperkeratosis/lichenification, vesiculation, oedema or fissures’ to the presence of ‘barely perceptible erythema’ and ‘no signs of scaling, hyperkeratosis/lichenification, vesiculation, oedema or fissures’. Following these adjustments, evaluation of the psychometric properties of the modified IGA–CHE was considered important to support its use as a clinical trial endpoint and for evaluating CHE severity in clinical practice.

The aim of this research was to evaluate the measurement properties of the IGA–CHE, a new clinician-reported assessment of the severity of CHE signs. This research also aimed to support interpretation of the IGA–CHE scores when used as an outcome assessment in clinical practice or to derive clinical trial endpoints, and ultimately support label claims, awarded by regulators.

## Methods

### Study design

Data from a phase 3, randomized, double-blind, vehicle-controlled, parallel-group, multi-site trial evaluating the efficacy and confirming the safety of delgocitinib cream in adult patients with moderate to severe CHE (ClinicalTrials.gov ID: NCT04871711) were used for these psychometric evaluation analyses. Patients were assigned to receive delogcitinib cream 20 mg/g or cream vehicle at a ratio of 2:1. Treatment was applied twice daily for 16 weeks. CHE severity was assessed at the trial site by a clinician at screening and then at Weeks 0, 1, 2, 4, 8, 12, and 16 using the IGA–CHE instrument. The primary endpoint was the proportion of patients achieving IGA–CHE treatment success, which was defined as clear (a score of ‘0’) or almost clear (‘1’) from Baseline to Week 16. An institutional review board at each study site approved the study protocol and all activities were conducted in compliance with the International Committee on Harmonization and applicable Good Clinical Practice standards and in accordance with the Declaration of Helsinki and its later amendments.

### Participant sample

Subjects were recruited from clinical sites in Canada, France, Germany, Italy, Poland, and the United Kingdom. To be eligible to participate, subjects were required to have a diagnosis of CHE, defined as HE that has persisted for more than 3 months or returned twice or more within the last 12 months, as well as moderate to severe CHE at screening and Baseline according to the IGA–CHE (score of 3 or 4) and a Hand Eczema Symptom Diary (HESD) Itch score [13] (weekly average) of ≥ 4 points for the 7 days preceding Baseline (Full eligibility criteria is provided in Supplemental Table 1). All participants provided written informed consent prior to the conduct of any study activities.

### Overview of measures

#### Investigator Global Assessment of Chronic Hand Eczema (IGA–CHE)

The IGA–CHE is a single item ClinRO that allows investigators to assess overall disease severity at one given timepoint and consists of a five-level severity scale (i.e., 0 = ‘clear’, 1 = ‘almost clear’, 2 = ‘mild’, 3 = ‘moderate’, 4 = ‘severe’) [6]. Each severity level on the scale is clinically characterized in terms of erythema, scaling, hyperkeratosis, vesiculation, oedema, and fissures (Table 1). Assessment is based on the condition of the subject’s disease at the time of evaluation and not in relation to the condition at a previous visit. New lesions on previously untreated areas were included in the assessment. The IGA–CHE for a specific visit is the raw score determined by the clinician.

**Table 1:** Composition of the IGA–CHE

IGA–CHE severity	IGA–CHE score	Sign and intensity	
Clear	0	No signs of erythema, scaling, hyperkeratosis/lichenification, vesiculation, oedema or fissures	
Almost clear	1	Barely perceptible erythema	
		No signs of scaling, hyperkeratosis/lichenification, vesiculation, oedema or fissures	
Mild	2	At least one:	And at least one:
		Slight but definite erythema (pink)	Scattered vesicles, without erosion
		Slight but definite scaling (mostly fine scales)	Barely palpable oedema
		Slight but definite hyperkeratosis/lichenification	Superficial fissures
Moderate	3	At least one:	And at least one:
		Clearly perceptible erythema (dull red)	Clustered vesicles, without visible erosion
		Clearly perceptible scaling (coarse scales)	Definite oedema
		Clearly perceptible hyperkeratosis/lichenification	Definite fissures
Severe	4	At least one:	And at least one:
		Marked erythema (deep or bright red)	High density of vesicles with erosions
		Marked and thick scaling	Marked oedema
		Marked hyperkeratosis/lichenification	One or more deep fissures

*IGA–CHE* Investigator Global Assessment of Chronic Hand Eczema

#### Convergent validity measures

Other clinician- and patient-reported outcome measures administered alongside the IGA–CHE were used to: a) support evaluation of convergent validity of the IGA–CHE; b) define patients with stable CHE for test–retest reliability analysis; and c) define subjects who experienced change, described in detail below.

*Patient Global Assessment of Disease Severity (PaGA)*. The PaGA is a patient-reported outcome (PRO) global assessment of disease severity in which patients rate their CHE severity on a five-level scale (0 = ‘clear’ [no hand eczema symptoms], 1 = ‘almost clear’ [only slight redness, no other hand eczema symptoms], 2 = ‘mild’, 3 = ‘moderate’, and 4 = ‘severe’) and is based on the severity of a patient’s HE at the time of assessment. The PaGA was completed on an electronic device at the trial site at Baseline and at Weeks 1, 2, 4, 6, 12, and 16.

*Hand Eczema Severity Index (HECSI)*. The HECSI is a ClinRO that clinicians use to rate the severity of six clinical signs of HE (erythema, infiltration/papulation, vesicles, fissures, scaling, and oedema) at the time of evaluation [14, 15]. The HECSI score is calculated by dividing the patient’s hand into five areas (fingertips, fingers, palms, back of hands, and wrists) and the intensity of each of the six clinical signs are measured, using a 4-point severity scale (0 = ‘none/absent’, 1 = ‘mild’, 2 = ‘moderate’, and 3 = ‘severe’). For each location, the area score (total of both hands) is calculated by assigning a score of 0–4 based on the following criteria: 0 = ‘0%’, 1 = ‘1–25%’, 2 = ‘26–50%’, 3 = ‘51–75%’, 4 = ‘76–100%.’ The score given for each location is multiplied by the total sum of the intensity of each clinical feature. Total score ranges from 0 to 360 with higher scores indicating greater severity of CHE. The HECSI was administered at Baseline and Weeks 1, 2, 4, 8, 12, and 16.

*Hand Eczema Symptom Diary Patient Global Impression of Severity (HESD PGI-S)*. The HESD PGI-S is a single item Patient-Global Impression of Severity designed to assess patients’ global perception of the severity of CHE signs and symptoms over the past week and using a 4-point categorical response scale (‘none’, ‘mild’, ‘moderate’, and ‘severe’). The HESD PGI-S was completed on an electronic device at the trial site at Baseline and Weeks 2, 4, 8, and 16.

*Hand Eczema Symptom Diary Patient Global Impression of Change (HESD PGI-C)*. The HESD PGI-C is a single item Patient-Global Impression of Change designed to assess patient perceptions of the overall change in their CHE signs and symptoms since starting the trial treatment, using a 5-point categorical response scale (‘much better’, ‘a little better’, ‘no change’, a little worse’, and ‘much worse’). The HESD PGI-C was completed on an electronic device at the trial site at Weeks 2, 4, 8, and 16.

### Statistical methods

Table 2 details the main statistical methods used in this study, designed to evaluate different aspects of IGA–CHE score performance. Other than the cross-tabulation of the IGA–CHE and PaGA, all statistical analyses were detailed a priori in a psychometric analysis plan, finalized prior to receiving the data. The psychometric analysis population, comprised of the first 280 subjects randomized with an IGA–CHE completion at Baseline and Week 16, was used to for all analyses unless otherwise specified. This consisted of a cut of the blinded phase 3 trial data, pooled across delgocitinib cream and cream vehicle groups. All analyses were performed by independent psychometricians not involved with the trial efficacy analyses. Psychometric evaluation was conducted in accordance with best practice guidance from regulators for assessing measurement properties of Clinical Outcome Assessments (COAs) [7–11].

**Table 2:** Summary of psychometric analyses in the phase 3 clinical trial

Analysis	Description
Test–retest reliability	Test–retest reliability evaluated consistency in scores between Weeks 2 and 4 and between Weeks 4 and 8 in a subset of subjects defined as having ‘stable’ CHE severity using other trial measures (detailed below). Test–retest reliability was evaluated through calculation of Cohen’s Kappa (*k*) coefficient with quadratic weighting for subjects defined as stable
	The following cutoffs were employed to interpret the kappa values: > 0.75 indicated excellent agreement, 0.40–0.75 indicated good–fair agreement, and > 0.40 indicated poor agreement [16]
	Stability was defined based on subjects with: No change on the PaGA between Weeks 2 and 4 No change on the PaGA between Weeks 4 and 8 No change on the HESD PGI-S between Weeks 2 and 4 No change on the HESD PGI-S between Weeks 4 and 8 Change on the HECSI of < 0.50 the Baseline Standard Deviation (SD) between Weeks 2 and 4 Change on the HECSI of < 0.50 the Baseline SD between Weeks 4 and 8
Convergent validity	Convergent validity of the IGA–CHE was evaluated using data collected at Week 4, by examining correlations with the PaGA, HESD PGI-S, and HECSI scores
	When evaluating convergent validity, score assessing similar or related concepts are expected to be at least moderately correlated. It was hypothesized that all of the above concurrent measures would correlate at ≥ 0.40 with the IGA–CHE [17]
	Correlation size was interpreted as: correlations of < 0.50 were defined a priori as ‘weak’, those ≥ 0.50 and < 0.70 as ‘moderate’, those ≥ 0.70 and < 0.90 as ‘strong’, and those ≥ 0.90 were considered ‘very strong’ [18]
	Week 4 was chosen for the convergent validity and known groups validity (see row below) analyses as it was expected that there would be a greater distribution of scores across the IGA–CHE scale than at Baseline (when trial inclusion criteria required that all subjects would be at the upper end of the response scale)
Known-groups validity	Construct validity was also assessed using the known-groups method to evaluate differences in scores among groups of patients who differ on variables hypothesized to influence the construct of interest
	Again, this analysis was performed at Week 4
	CHE severity groups for comparison were defined by responses to the PaGA (comparison of patients scoring: 0–1 = ‘clear or almost clear’, 2 = ‘mild’, 3 = ‘moderate’, and 4 = ‘severe’) and HESD PGI-S (comparison of patients scoring: ‘none’, ‘mild’, ‘moderate’, and ‘severe’)
	The magnitude of differences between the groups was evaluated using between-group effect size estimates, calculated using the pooled standard deviation as the denominator, and based on the differences between each adjacent pair of groups [19]. Use of the pooled SD assumed both groups have similar variance
	The following cutoffs were used to interpret the magnitude of each effect size: small difference = 0.20, moderate difference = 0.50, large difference = 0.80 [20]
	*F* test calculated by one-way ANOVA (comparison of more than two groups) and Fisher’s exact test were used to evaluate if differences among the groups were statistically significant (*p* ≤ 0.05)
Ability to detect change	Ability to detect change assesses whether a score fluctuates in line with true change in the construct it measures
	Changes in IGA–CHE scores from baseline to Week 16 were compared between groups defined as ‘improved’, ‘stable’, and ‘worsened’ based on changes in PaGA, HESD PGI-S, HESD PGI-C, and HECSI scores
	Within- and between-group effect sizes and between groups one-way ANOVA *F* test were calculated to evaluate the magnitude and significance of the differences in change scores within and between these groups, respectively
	Patients were categorized into ‘improved’, ‘stable’, and ‘worsened’ as follows:
	PaGA and HESD PGI-S Improved: ≥ 1 grade improvement Stable: no change Worsened: ≥ 1 grade worsening HESD PGI-C Improved: ‘a little better’ or ‘much better’ Stable: ‘no change’ Worsened: ‘a little worse’ or ‘much worse’ HECSI Improved: subjects who have a HECSI improvement ≥ 0.50 Baseline SD Stable: subjects who have a HECSI change score < 0.50 Baseline SD Worsened: subjects who have a HECSI worsening ≥ 0.50 Baseline to SD
	The between-group effect sizes were calculated and interpreted as described for the known groups. Within-group effect sizes [21] were calculated as the mean change score divided by the SD of the score at the earlier of the two timepoints. The same thresholds as in known groups were again used to interpret the changes within groups and differences in changes between groups
Interpretation of scores: anchor-based analyses to inform within-subject meaningful change thresholds	Score interpretation characterizes how meaning is attributed to observed changes and differences in scores, beyond that provided for by statistically significant results
	In anchor-based approaches to defining meaningful change thresholds, an external indicator is used to identify subjects who have experienced an improvement in the concept being measured
	The suitability of the proposed anchors was tested by examining the correlation of the change in anchor and change in IGA–CHE scores. Anchors with correlations of < 0.30 were not taken forward for analysis [22].
	Thresholds for within-subject and between groups meaningful change were estimated by calculating the mean changes in IGA–CHE scores for subjects classified as ‘moderately improved’ or ‘minimally improved’ based on the following anchors: PaGA, HESD PGI-S, HESD PGI-C, HECSI-75 (subjects who improved in their HECSI scores by 75%), and HECSI-90 (subjects who improved in their HECSI scores by 90%)
	Estimates were plotted on a forest plot to visualise the range of estimates and identify a plausible range of values for meaningful change
	A correlation weighted average with Fisher’s Z transformation (considering the strength of each anchors’ correlation with the target score) was used to identify a single value [23]
	Analyses were conducted for change from Baseline to Week 16
	In addition, a cross-tabulation of IGA–CHE scores with PaGA scores was performed at Baseline, Week 8 and Week 16 to further aid score interpretation; this was the only post-hoc analysis
Interpretation of scores: distribution-based analyses	In addition to the anchor-based analyses, distribution-based analyses involved using the distributional properties of the IGA–CHE score to provide an indication of the amount of change beyond measurement error that may be considered meaningful
	This involved calculation of 0.5 of the SD at Baseline and the standard error of measurement (SEM) [24, 25]. The SEM was calculated as the SD at Baseline multiplied by the square root of one minus the reliability of the score at Baseline [SD × (1 – *r*)1/2]. The Kappa coefficient calculated within the test–retest analyses using the HESD PGI-S anchor (Weeks 2–4) was used for the reliability coefficient. A value of ‘1 SEM’ was used as the estimate of the meaningful change threshold

*ANOVA* analysis of variance, *CHE* Chronic Hand Eczema, *HECSI* hand eczema severity index, *HESD PGI-C* hand eczema symptom diary patient global impression of change, *HESD PGI-S* hand eczema symptom diary patient global impression of severity, *IGA–CHE* Investigator Global Assessment of Chronic Hand Eczema, *PaGA* patient global assessment of disease severity

## Results

### Sample characteristics

Key demographic and clinical characteristics are provided in Table 3. The sample included more female (65.7%) than male subjects and most were white/Caucasian (88.2%) and clinically classified as Fitzpatrick skin types II or III (43.2% and 41.1%, respectively).

**Table 3:** Demographic and clinical characteristics for the psychometric analysis population at Baseline

Description	Psychometric analysis population (*N* = 280)
Gender—*n* (%)	
Female	184 (65.7%)
Male	96 (34.3%)
Age	
*n*	280
Mean (SD)	43.3 (14.3)
Median	44
Min, Max	19, 77
Race—*n* (%)	
American Indian or Alaska Native	1 (0.4%)
Asian	12 (4.3%)
Black or African American	2 (0.7%)
White	247 (88.2%)
Multiple	1 (0.4%)
Not Reported	16 (5.7%)
Other	1 (0.4%)
Ethnic origin—*n* (%)	
Hispanic or Latino	10 (3.6%)
Not Hispanic or Latino	255 (91.1%)
Not reported	15 (5.4%)
Fitzpatrick skin type—*n* (%)	
Type I	14 (5.0%)
Type II	121 (43.2%)
Type III	115 (41.1%)
Type IV	26 (9.3%)
Type V	3 (1.1%)
Type VI	1 (0.4%)
CHE severity—*n* (%)	
3—Moderate	189 (67.5%)
4—Severe	91 (32.5%)
HECSI total score	
*n*	280
Mean (SD)	80.6 (52.3)

Psychometric analysis population defined as the first 280 subjects randomised

Some countries such as France are not allowed to report ethnicity and race data, where this is the case the ‘Not reported’ option was used, this is different to missing data

*SD* Standard Deviation, *HECSI* Hand Eczema Severity Index

### Test–retest reliability

The IGA–CHE demonstrated ‘good’ test–retest reliability (kappa coefficients = 0.63–0.69) when subjects were defined as stable based on the PaGA, HESD PGI-S, and HECSI between Weeks 2 and 4. Test–retest reliability was ‘excellent’ (kappa coefficient = 0.76 for all analyses) when subjects were defined as stable on the same measures between Weeks 4 and 8 (Table 4).

**Table 4:** IGA–CHE score weighted Kappa coefficient (k) estimates of test–retest reliability

Anchor for defining stability of CHE severity	Timepoint	*n* (%)^a^	K Estimate^b^ (95% CI)
No change on the PaGA	Weeks 2–4	142 (50.7%)	0.63 (0.53, 0.73)
	Weeks 4–8	144 (51.4%)	0.76 (0.69, 0.83)
No change on the HESD PGI-S	Weeks 2–4	154 (55.0%)	0.68 (0.59, 0.76)
	Weeks 4–8	161 (57.5%)	0.76 (0.69, 0.83)
< 0.50 Baseline SD on the HECSI	Weeks 2–4	218 (77.9%)	0.69 (0.62, 0.78)
	Weeks 4–8	227 (81.1%)	0.76 (0.70, 0.81)

*CHE* Chronic Hand Eczema, *CI* confidence interval, *PaGA* patient global assessment of disease severity, *HESD PGI-S* hand eczema symptom diary patient global impression of severity, *HECSI* hand eczema severity index

^a^*n* represents the number of subjects who are stable with regards to the anchor measure

^b^*k* Estimate Kappa coefficient (*k*) with quadratic weighting is used as data are ordered categorical on a single item measure

### Convergent validity

Correlations were examined between the IGA–CHE scores and the PaGA and HESD PGI-S at Week 4 (Table 5). All correlations were moderate or strong (range: 0.65–0.72) and exceeded the hypothesized minimum threshold, providing strong evidence of convergent validity.

**Table 5:** Correlation of IGA–CHE scores with convergent measures at Week 4

Measure^a^	*n*^b^	Polychoric correlation coefficients	Polyserial correlation coefficients
PaGA	271	0.72	–
HESD PGI-S	271	0.65	–
HECSI	272	–	0.68

*PaGA* patient global assessment of disease severity, *HESD PGI-S* hand eczema symptom diary patient global impression of severity, *HECSI* hand eczema severity index

^a^All measures are scored, so that higher scores mean worse Chronic Hand Eczema severity

^b^*n* represents the number of subjects in the psychometric analysis population without form level missing data at week 4

### Known-groups validity

IGA–CHE scores were compared among groups who differed in their CHE severity as reported on the PaGA and HESD PGI-S (Table 6). There was a pattern of significantly higher mean IGA–CHE scores (indicating worse CHE severity) for subjects who also scored higher (worse) on the PaGA and HESD PGI-S (*p* < 0.001), with the expected monotonic increases across severity groups. Effect sizes between adjacent groups were moderate to large (ES > 0.71), except for the comparison between the HESD PGI-S “mild” group and “none” group, which had a small effect size (ES = 0.44), but only just below the threshold for moderate. These results provide strong evidence regarding the ability of the IGA–CHE to distinguish patients of clear/almost clear, mild, moderate, and severe severity levels, supporting the construct validity of the IGA–CHE score.

**Table 6:** Known groups validity for the IGA–CHE scores at Week 4

Item/Score Anchor	*n*^a^	Mean IGA–CHE score (SD)	Median IGA–CHE score	Between groups effect size^b^	*p* value^c^
PaGA					
Responses 0–1: clear–almost clear [reference group]	45	1.5 (0.76)	2.0		< 0.001
Response 2: mild	94	2.2 (0.67)	2.0	0.98	
Response 3: moderate	100	2.8 (0.70)	3.0	0.76	
Response 4: severe disease	32	3.4 (0.56)	3.0	0.98	
HESD PGI-S					
None [reference group]	16	1.7 (0.87)	2.0		< 0.001
Mild	123	2.0 (0.72)	2.0	0.44	
Moderate	99	2.8 (0.70)	3.0	1.11	
Severe disease	33	3.3 (0.68)	3.0	0.71	

*IGA–CHE* investigator global assessment of severity of Chronic Hand Eczema, *PaGA* patient global assessment of disease severity, *HESD PGI-S* hand eczema symptom diary patient global impression of severity, *SD* standard deviation

^a^*n* represents the number of subjects in the psychometric analysis population without form level missing data at week 4

^b^Calculated using Hedge’s g between adjacent groups. Hedge’s g is calculated as the difference in means divided by the pooled standard deviation

^c^The statistical significance (*p* ≤ 0.05) of differences in scores between groups was calculated using the *F* test of one-way ANOVAs

Cross-tabulated tables of categorical IGA–CHE and PaGA scores at Week 8 and Week 16 provide further evidence of known-groups validity. Tables 7 and 8 show these cross-tabulations collapsed in line with the endpoint categories in the phase 3 trial. These results show higher frequencies, where the response options are the same for IGA–CHE and PaGA at Week 8, indicating subjects who scored more severely on the PaGA also scored more severely on the IGA–CHE and vice versa. At Week 16, this is seen for the severe/moderate/mild aligned responses, but a higher frequency of subjects was observed in the PaGA almost clear/clear group with IGA–CHE severe/moderate/mild (*n* = 40), compared to the almost clear/clear aligned groups (*n* = 30). However, a low frequency was observed for the PaGA severe/moderate/mild with IGA–CHE almost clear/clear group (*n* = 7). This indicates subjects scored more severely on the IGA–CHE than the PaGA later in the treatment period. This suggests that the clinicians were rating the patients slightly more severely than patients were rating themselves, perhaps because in the IGA–CHE clinicians base their rating on observable signs, whereas patients will consider pain and itch, which are not observable.

**Table 7:** Cross-tabulations of IGA–CHE and PaGA scores at Week 8

	PaGA		
IGA–CHE	Severe/moderate/mild, *n*	Almost clear/clear, *n*	Total, *n*
Severe/moderate/mild, *n*	193	28	221
Almost clear/clear, *n*	18	32	50
Total, *n*	211	60	271

**Table 8:** Cross-tabulations of IGA–CHE and PaGA scores at Week 16

	PaGA		
IGA–CHE	Severe/moderate/mild, *n*	Almost clear/clear, *n*	Total, *n*
Severe/moderate/mild, n	192	40	232
Almost clear/clear n	7	30	37
Total n	199	70	269

### Ability to detect change

Changes in IGA–CHE scores were compared among subjects defined as “improved”, “stable”, and “worsened” on the PaGA, HESD PGI-S, HESD PGI-C, and HECSI between Baseline and Week 16. These results provide evidence that the IGA–CHE can detect change over time, regardless of the rating used to define change. As shown in Table 9, the IGA–CHE score was able to detect improvement, with large effect sizes (ES ≥ 2.79) for the improved group for all anchors. In all cases, the effect size for the stable group was smaller than the improved group, with moderate to large within-group effect sizes (ES range – 0.60 to – 1.10). Differences between change groups were statistically significant (*p* < 0.001), and between-group effect sizes were large (ES ≥ 1.00) between those defined as improved and stable subjects. Results for the subjects categorized as worsening only showed small changes; however, the sample sizes for the PaGA, HESD PGI-S, and HECSI worsened groups were small (*n* ≤ 15). The results provide strong evidence for the ability of the IGA–CHE to detect improvement.

**Table 9:** IGA–CHE ability to detect change between Baseline and Week 16

Grouping variable	*n*	Mean change score (SD)	Median change score (Min–Max)	Within groups effect size^a^	Between groups effect size^b^	Between groups *p* value^c^
PaGA						
≥ 1 category improvement	184	– 1.3 (0.98)	– 1.0 (– 4, 1)	– 2.86	– 1.11	< 0.001
Change score = 0	65	– 0.3 (0.62)	0.0 (– 2, 1)	– 0.72		
≥ 1-level worsening	15	– 0.2 (0.68)	0.0 (– 1, 1)	– 0.43	0.22	
HESD PGI-S						
≥ 1 category improvement	189	– 1.3 (0.97)	– 1.0 (– 4, 1)	– 2.79	– 1.04	< 0.001
Change score = 0	62	– 0.4 (0.73)	0.0 (– 3, 1)	– 0.76		
≥ 1-level worsening	11	– 0.1 (0.54)	0.0 (– 1, 1)	– 0.19	0.38	
HESD PGI-C						
‘A little better’ + ‘Much better’	186	– 1.4 (0.96)	– 1.0 (– 4, 1)	– 2.91	– 1.20	< 0.001
‘No change’	46	– 0.3 (0.62)	0.0 (– 1, 1)	– 0.60		
‘A little worse’ + ‘Much worse’	37	– 0.2 (0.75)	0.0 (– 2, 1)	– 0.46	0.10	
HECSI						
≥ 0.50 Baseline SD improvement	168	– 1.4 (0.94)	– 1.0 (– 4, 1)	– 3.02	– 1.00	< 0.001
< 0.50 SD change	103	– 0.5 (0.84)	0.0 (– 3, 1)	– 1.10		
≥ 0.50 Baseline SD worsening	9	0.1 (0.93)	0.0 (– 1, 1)	0.24	0.74	

*PaGA* patient global assessment of disease severity, *HESD PGI-S* hand eczema symptom diary patient global impression of severity, *HESD PGI-C* hand eczema symptom diary patient global impression of change, *HECSI* hand eczema severity index, *SD* standard deviation

^a^Effect size is calculated as mean change score divided by the SD of the score at the earlier of the two timepoints

^b^Between groups effect size was calculated using Hedge's g between adjacent groups. Hedge's g is calculated as the difference in means divided by the pooled standard deviation

^c^Statistical significance of any differences in change scores between the three groups were examined using one-way ANOVA *F* tests. *p* values ≤ 0.05 are considered statistically significant

### Interpretation of scores

Correlations between changes in the IGA–CHE and conceptually similar measures (i.e., PaGA, HESD PGI-S, HESD PGI-C, HECSI-75, and HECSI-90) were all moderate or strong (> 0.50), indicating they are adequately related to support meaningful change analyses. To inform the most appropriate responder definition for the IGA–CHE, the moderately improved group was defined a priori as the group of primary interest for all anchors, except the HESD PGI-C, where the minimally improved group was of primary interest (there was no change level equivalent to moderate improvement due to the HESD PGI-C response scale). As the HECSI-75 and HECSI-90 only have one improvement group counted as responders, this was used as the primary interest group for these anchors.

**Fig. 1:**
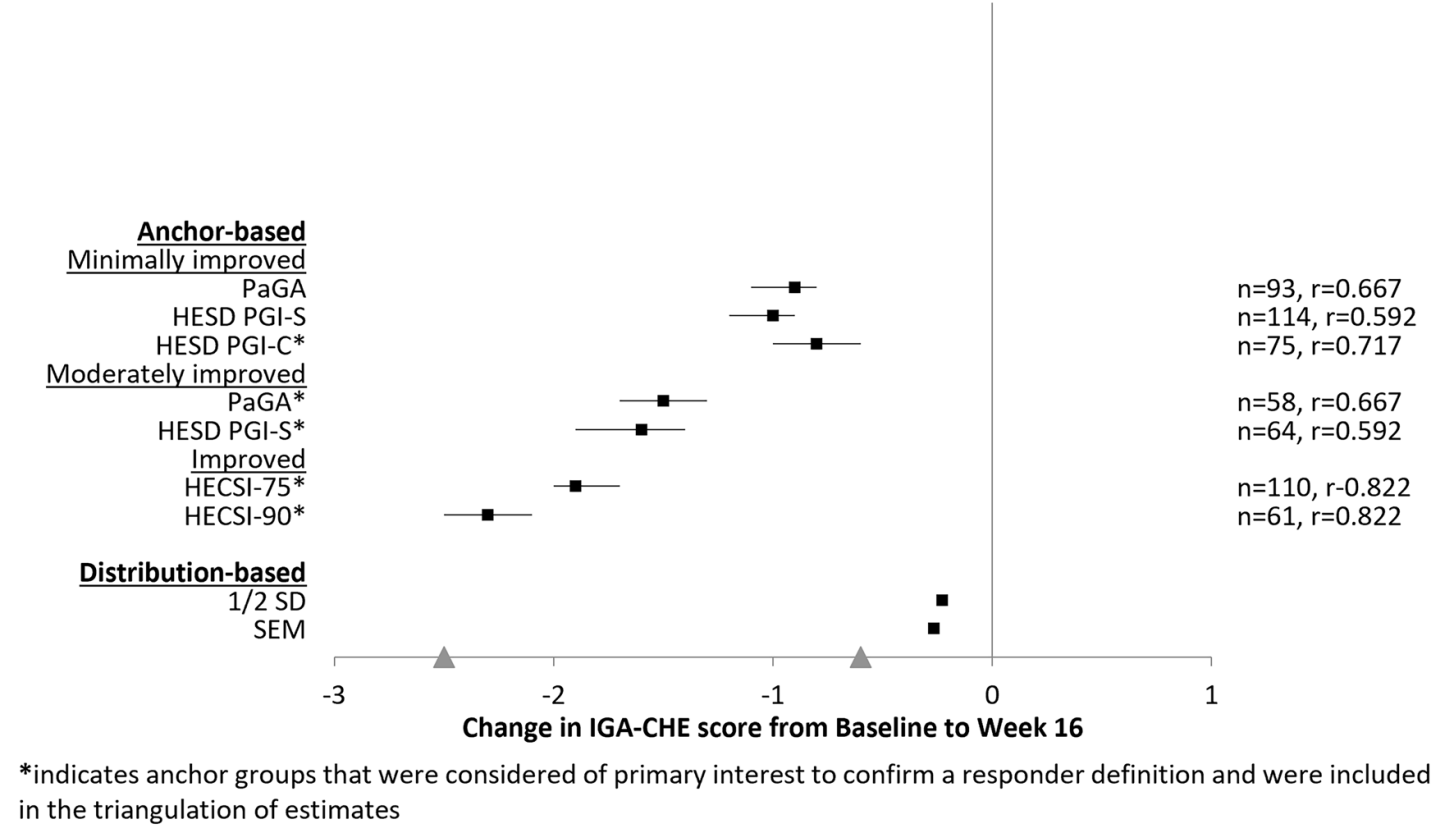
Forest plot showing within-group mean change and distribution-based meaningful change estimates for the IGA–CHE

Subjects in the psychometric analysis population who had a minimal improvement on the anchors had mean IGA–CHE score changes between – 0.8 and – 1.0, and subjects who had a moderate improvement on the anchors had mean IGA–CHE score changes between – 1.5 and – 1.6. For the anchor groups of primary interest, subjects had IGA–CHE improvements between – 0.8 and – 2.3 (see Fig. 1). A correlation weighted average with Fisher’s z transformation (accounting for the strength of each anchor’s correlation with the target score) suggested a single value of – 1.7. However, only 2-level or 1-level change is possible for an individual on the IGA–CHE due to the categorical response scale. Empirical Cumulative Distribution (eCDF) and Probably Density Function (PDF) curves supported further examination of both 1-level change and 2-level change as possible responder definitions.

**Fig. 2:**
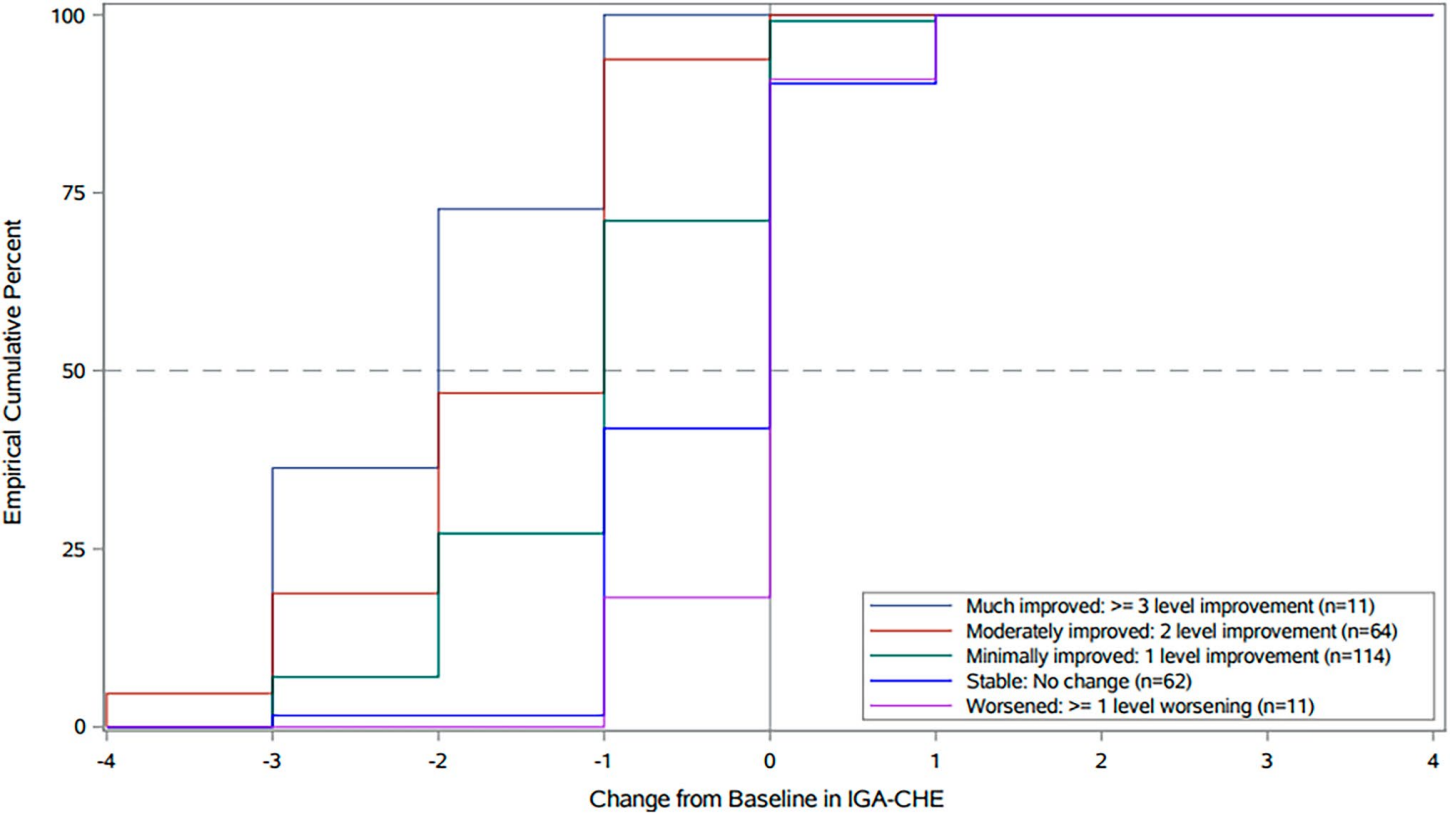
eCDF of IGA–CHE change from Baseline scores by HESD PGI-S group at Week 16

The eCDF and PDF curves showed separation of patients considered minimally improved and moderately improved on the anchors from those considered stable with both a 1-level and 2-level change (see Fig. 2 as an example; the remaining eCDF curves are available in the online supplementary material). Thus, results provide support for both 1-level and 2-level improvement on the IGA–CHE as being appropriate thresholds for defining within-patient clinically meaningful change (noting that a whole level change is required for within-subject change thresholds considering the ordinal nature of the scale). It is suggested that when there is a preference for taking a relatively conservative approach to be very confident of meaningful treatment benefit, a 2-level change on the IGA–CHE can be used as the threshold. As Fig. 2 shows, a 2-level IGA–CHE change would classify < 10% stable subjects as improved according to the HESD PGI-S but also < 50% of moderately improved subjects as improved suggesting it is a high threshold. However, findings also provide support for a 1-level change being important and meaningful to patients. As Fig. 2 shows, a 1-level IGA–CHE change would classify approximately 90% of moderately improved subjects as improved according to the HESD PGI-S, but also approximately 40% of subjects who are stable as improved. Importantly, a 1-level change was well above the distribution-based estimates, indicating that this level of change is above measurement error.

Cross-tabulated ordinal change summaries of IGA–CHE and HESD PGI-S scores support these findings. Table 10 shows that 46.9% of subjects who achieved a 2-level improvement on the HESD PGI-S achieved only a 1-level improvement on the IGA–CHE; 46.9% also a achieved a ≥ 2-level change on the IGA–CHE (summing those with a 2-level improvement on the HESD PGI-S and 2-level, 3-level or 4-level improvement on IGA–CHE). These data provide further evidence that a 2-level change threshold on IGA–CHE is a relatively conservative responder threshold, as less subjects were able to achieve improvement according to this definition compared to the anchor.

**Table 10:** Cross-tabulated ordinal summary of IGA–CHE change by HESD–PGI-S scores from Baseline to Week 16

	Change on the HESD PGI-S				
	≥ 3-level improvement	2-level improvement	1-level improvement	No change	≥ 1-level worsening
IGA–CHE change					
4-level improvement	0 (0%)	3 (4.7%)	0 (0%)	0 (0%)	0 (0%)
3-level improvement	4 (36.4%)	9 (14.1%)	8 (7.0%)	1 (1.6%)	0 (0%)
2-level improvement	4 (36.4%)	18 (28.1%)	23 (20.2%)	0 (0%)	0 (0%)
1-level improvement	3 (27.3%)	30 (46.9%)	50 (43.9%)	25 (40.3%)	2 (18.2%)
No change	0 (0%)	4 (6.3%)	32 (28.1%)	30 (48.4%)	8 (72.7%)
1-level worsening	0 (0%)	0 (0%)	1 (0.9%)	6 (9.7%)	1 (9.1%)
2-level worsening	0 (0%)	0 (0%)	0 (0%)	0 (0%)	0 (0%)
3-level worsening	0 (0%)	0 (0%)	0 (0%)	0 (0%)	0 (0%)
4-level worsening	0 (0%)	0 (0%)	0 (0%)	0 (0%)	0 (0%)

*IGA–CHE* Investigator Global Assessment of Chronic Hand Eczema, *HESD PGI-S* Hand eczema symptom diary patient global impression of severity

## Discussion

The aim of this study was to psychometrically evaluate the IGA–CHE to support its use as a clinical trial endpoint as well as in clinical practice for assessing CHE severity. Analyses were performed in accordance with best practices for assessing measurement properties of COAs [7–11]. Findings provide strong evidence supporting the psychometric validity of the IGA–CHE as a comprehensive single-item measure of CHE severity that is reliable and valid, quick and easy to administer, discriminates between patients of differing CHE severity levels and is sensitive to changes in severity over time.

Test–retest reliability results met the threshold for moderate or excellent agreement across the timepoints assessed and regardless of how stability was defined. Although the timepoints used (i.e., 2 and 4 weeks, respectively) are arguably relatively long to expect CHE signs to remain stable, the strength of these results suggest that if it were feasible to examine test–retest over a shorter timeframe the results would be at least equally strong.

Strong or moderate correlations with other measures of related concepts (i.e., HECSI, PaGA and HESD PGI-S), provide evidence of convergent validity and that the IGA–CHE is truly measuring CHE sign severity. Similarly, known groups comparisons showed that the IGA–CHE can distinguish groups of patients who differ in CHE severity on other measures, with statistically significant differences among those groups. The IGA–CHE was also shown to be sensitive to improvements in CHE severity, with large effect sizes within groups defined as ‘improved’ and large between-group differences between ‘improved’ and ‘stable’ groups.

Evidence generated from the anchor-based analyses supports a 2-level change in IGA–CHE scores as a conservative threshold for defining within-subject clinically meaningful change (derived from the – 1.7 single value). Nonetheless, because the analyses suggest a meaningful change threshold could lie anywhere between – 0.8 and – 2.3, the findings also provide support that 1-level change can also be considered an appropriate meaningful change threshold. This was further supported by the distribution-based analyses.

Another example of a single item measure of a patient’s overall CHE severity is the Physician Global Assessment (PGA) used in the alitretinoin studies [26]. Although both IGA–CHE and PGA include five levels, representing 0 = 'clear', 1 = 'almost clear', 2 = 'mild', 3 = 'moderate', 4 = 'severe disease', they differ in that the IGA–CHE uses detailed descriptions within a single scale to characterize each level, whereas for the PGA these levels are defined based on assessment of the severity of each sign or symptom using a separate outcome measure and photo guide. The descriptions for the IGA–CHE were defined carefully, with input from clinical experts and taking account of regulatory feedback, to ensure adjacent levels, in particular ‘almost clear’ and ‘mild’, are clearly distinct. For example, ‘almost clear’ in the IGA–CHE is defined as ‘barely perceptible erythema’ only, whereas definitions of ‘almost clear’ in PGA are broader which may make it more difficult to differentiate between adjacent levels, and potentially lead to inconsistent interpretation. As described above, the known groups findings reported here provide strong evidence this has resulted in a measure that discriminates well between clear/almost clear, mild, moderate, and severe severity levels.

We recognize some limitations in our study. Due to the sample being mainly Canadian and northern European, subjects were predominantly white/Caucasian. Future confirmation of psychometric validity in more racially and ethnically diverse populations would be of value. Furthermore, all psychometric evaluation to-date has been performed in a clinical trial sample. If the IGA–CHE is to be used in real-world studies or in general clinical practice, further evaluation in a ‘real-world’ sample would be beneficial to confirm the measurement properties are fully generalizable in all circumstances.

## Conclusion

The IGA–CHE is fit-for-purpose as a valid, reliable, and responsive measure of CHE severity that can be used to support clinical trial endpoints. The IGA–CHE also has value for use in clinical settings to assess CHE severity and monitor clinically meaningful changes in CHE severity over time or in response to treatment.

### Supplementary Information

Below is the link to the electronic supplementary material.

Supplementary file1 (DOCX 497 KB)

## Data Availability

The data sets are available from the corresponding author on reasonable request.
