# Supplementary material for: Validation of the Investigator Global Assessment of Chronic Hand Eczema (IGA–CHE): a new clinician reported outcome measure of CHE severity
Source: Arch Dermatol Res. 2024 Mar 20;316(4):110. doi: 10.1007/s00403-024-02818-3 (PMC10955004; doi:10.1007/s00403-024-02818-3)
Supplement: Supplementary file 1 — Supplementary file1 (DOCX 497 KB) [file 403_2024_2818_MOESM1_ESM.docx]

**Title:** Validation of the Investigator Global Assessment of Chronic Hand Eczema (IGA-CHE): a new clinician reported outcome measure of CHE severity

**Journal:** Archives of Dermatological Research

**Corresponding author**: Johnathan I Silverberg, School of Medicine and Health Sciences, The George Washington University, Washington, DC, USA (email: [jonathanisilverberg@gmail.com](mailto:jonathanisilverberg@gmail.com))

| Supplemental Table 1. Eligibility criteria for the phase 3 clinical trial |
| --- |
| Inclusion criteria |
| 1. Signed and dated informed consent has been obtained prior to any protocol-related procedures. |
| 1. Aged 18 years or above at screening. |
| 1. Diagnosis of CHE, defined as hand eczema that has persisted for more than three months or returned twice or more within the last 12 months. |
| 1. Disease severity graded as moderate to severe at screening and Baseline according to IGA-CHE (i.e., an IGA-CHE score of 3 or 4) |
| 1. HESD Itch score (weekly average) of ≥4 points at Baseline. The Baseline weekly average was calculated from daily assessments of itch severity during the seven days immediately preceding the Baseline visit (Day -7 to Day -1). A minimum of four itch scores out of the seven days was required to calculate the Baseline average score. |
| 1. Subjects who had a documented recent history of inadequate response to treatment with TCS (at any time within one year before the screening visit) or for whom TCS were documented to be otherwise medically inadvisable (e.g., due to important side effects or safety risks).    - Inadequate response was defined as a history of failure to achieve and maintain a low disease activity state (comparable to an IGA-CHE score of ≤2) despite treatment with a daily regimen of TCS of class III-IV (potent to very potent) for Europe and class IV-I (medium potency to very/ultra-high potency) for Canada, applied at least 28 days or for the maximum duration recommended by the product prescribing information, whichever was shorter.    - Important side effects or safety risks were those that outweigh the potential treatment benefits and include intolerance to treatment, hypersensitivity reactions, and significant atrophy as assessed by the physician. |
| 1. Subjects adhered to standard non-medicated skin care including avoidance of known and relevant irritants and allergens. |
| 1. A women of childbearing potential who used an acceptable method of birth control throughout the trial up until the last application of IMP. |
| Exclusion criteria |
| 1. Concurrent skin diseases on the hands, e.g., tinea manuum. |
| 1. Active AD requiring medical treatment in regions other than the hands and feet. |
| 1. Active psoriasis on any part of the body. |
| 1. Hyperkeratotic hand eczema in combination with a history of psoriasis on any part of the body. |
| 1. Clinically significant infection (e.g., impetiginized hand eczema) on the hands. |
| 1. Systematic treatment with immunosuppressive drugs (e.g., methotrexate, cyclosporine, azathioprine), immunomodulating drugs, retinoids (e.g., alitretinoin), or corticosteroids within 28 days prior to Baseline (steroid eyedrops and inhaled or intranasal steroids corresponding to up to 1 mg prednisolone for allergic conjunctivitis, asthma, or rhinitis are allowed). |
| 1. Use of tanning beds, phototherapy (e.g., UVB, UVA1, PUVA), or bleach baths on the hands within 28 days prior to Baseline. |
| 1. Previous or current treatment with JAK inhibitors (including delgocitinib/LEO 124249), systemic or topical. |
| 1. Cutaneously applied treatment with immunomodulators (e.g., PDE-4 inhibitors, pimecrolimus, tacrolimus) or TCS on the hands within 24 days prior to Baseline. |
| 1. Use of systemic antibiotics or cutaneously applied antibiotics on the hands within 14 days prior to Baseline. |
| 1. Other transdermal or cutaneously applied therapy on the hands (except for the use of subject’s own emollients) within 7 days prior to Baseline. |
| 1. Cutaneously applied treatments in regions other than the hands, which could interfere with clinical trial evaluations or pose a safety concern within 7 days prior to Baseline. |
| 1. Treatment with any marketed biological therapy or investigational biologic agents (including immunoglobulin, anti-IgE, and dupilumab:    - Any cell-depleting agents including but not limited to rituximab: within 6 months prior to Baseline, or until lymphocyte count returns to normal, whichever was longer.    - Other biologics: within three months or five half-lives, whichever was longer, prior to Baseline. |
| 1. Treatment with any non-marketed drug substance (that is, an agent that has not yet been made fully available for clinical use following registration) within the last 28 days prior to Baseline or five half-lives, whichever is the longest. |
| 1. Clinically significant infection within 28 days prior to Baseline which, in the opinion of the investigator, may compromise the safety of the subject in the trial, interfere with evaluation of the IMP, or reduce the subject’s ability to participate in the trial. Clinically significant infections are defined as:  - A systemic infection. - A serious skin infection requiring parenteral (intravenous or intramuscular) antibiotics, antiviral, or antifungal medication. |
| 1. History of any known immunodeficiency disorder including a positive HIV virus test at screening, or the subject taking antiretroviral medication as determined by medical history and/or subject’s verbal report. |
| 1. Major surgery within 8 weeks prior to screening or planned in-patient surgery or hospitalization during the trial period. |
| 1. History of cancer:  - Subjects who have had basal cell carcinoma, localized squamous cell carcinoma of the skin or in situ carcinoma of the cervix were eligible provided that the subject was in remission and curative therapy was completed at least 12 months prior to screening. - Subjects who have had other malignancies are eligible provided that the subject was in remission and curative therapy was completed at least 5 years prior to screening. |
| 1. Any disorder which was not stable and:    - Affected the safety of the subject throughout the trial.    - Impeded the subjects ability to complete the trial.   Examples included but were not limited to cardiovascular, gastrointestinal, hepatic, renal, neurological, musculoskeletal, infectious, endocrine, metabolic, haematological, immunological, and psychiatric disorders, and major physical impairment. |
| 1. Any abnormal findings which:    - Put the subject at risk because of their participation in the trial.    - Influenced the subject’s ability to complete the trial   The abnormal finding was clinically significant and observed during the screening period. Examples included abnormal findings in physical examination, vital signs, ECG, haematology, clinical chemistry, or urinalysis. |
| 1. Positive hepatitis B surface antigen or hepatitis C virus antibody serology at screening. |
| 1. ALT or AST level ≥2.0×ULN at screening. |
| 1. Known or suspected hypersensitivity to any component(s) of the IMP. |
| 1. Current participation in any other interventional clinical trial. |
| 1. Previously randomized in this clinical trial. |
| 1. Current or recurrent chronic alcohol or drug abuse, or any other condition associated with poor compliance as judged by the investigator. |
| 1. Employees of the trial site, or any other individuals directly involved in the planning or conduct of the trial, or immediate family members of such individuals. |
| 1. Subjects who were legally institutionalized. |
| 1. Women who were pregnant or lactating. |


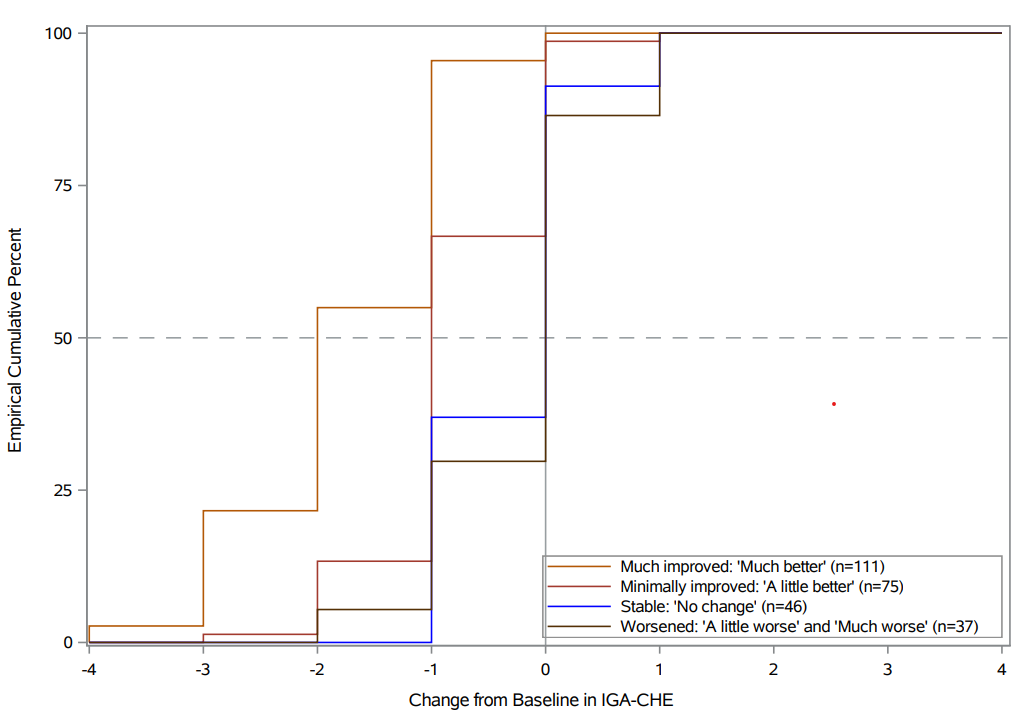


**Supplemental Figure 1. eCDF of IGA-CHE change from Baseline scores by HESD PGI-C group at Week 16**


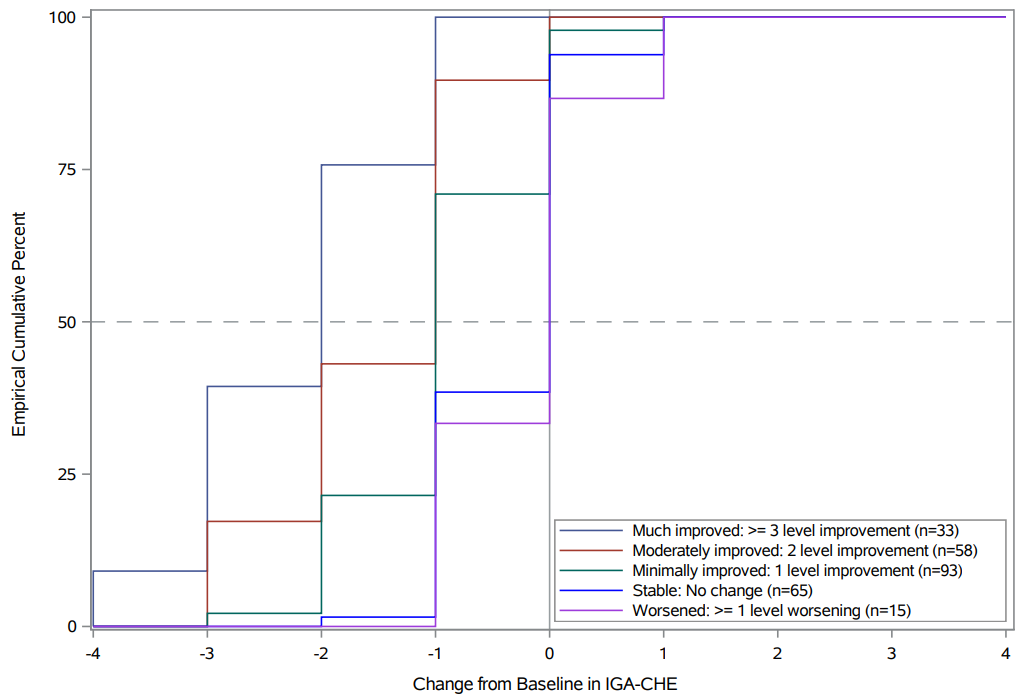


**Supplemental Figure 2. eCDF of IGA-CHE change from Baseline scores by PaGA group at Week 16**


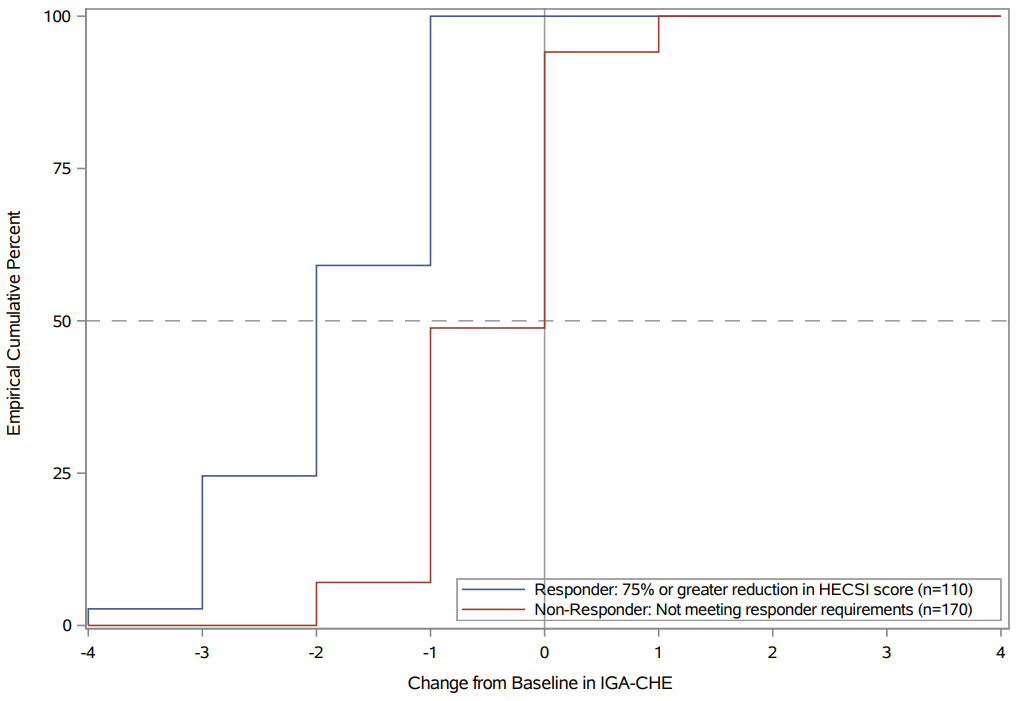


**Supplemental Figure 3. eCDF of IGA-CHE change from Baseline scores by HECSI-75 group at Week 16**


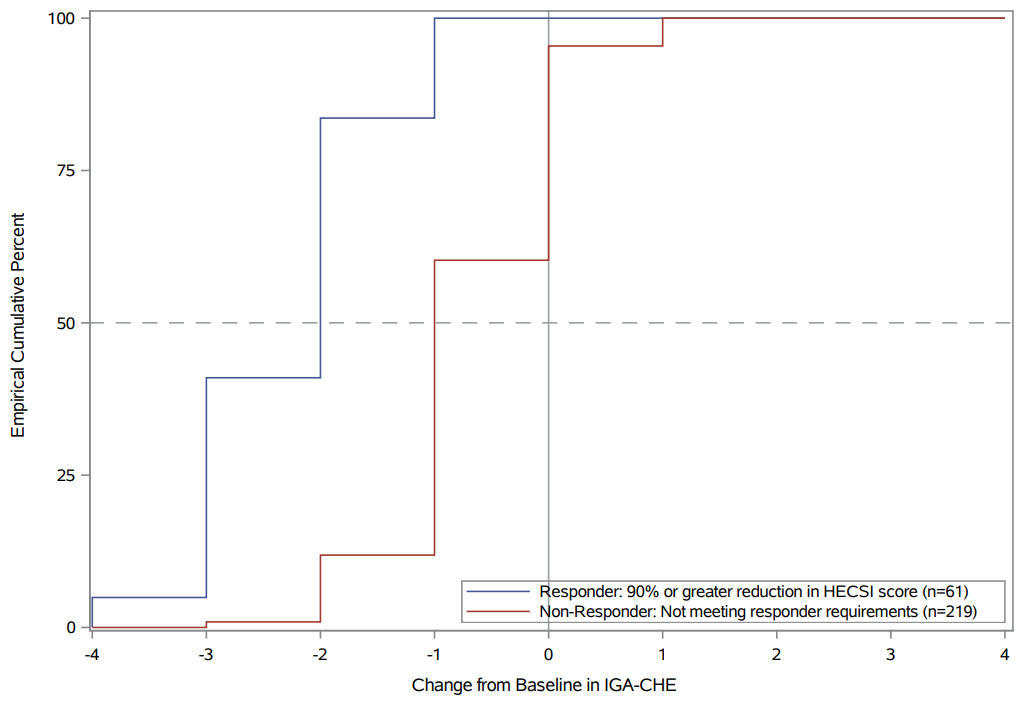


**Supplemental Figure 4. eCDF of IGA-CHE change from Baseline scores by HECSI-90 group at Week 16**
